# 

**Published:** 1901-04-27

**Authors:** 


**Notices of Births, Deaths, and Marriages** are inserted in this column at a charge of 2s. 6d. for an announcement not exceeding four lines.

### MARRIAGE.

Saxon-Farmborough—April 17th. at Holy Trinity Church, Bournemouth, by the Rev. G. W. Crock, M.A., assisted by the bride's father, Julius Conrau, fourth son of the late Samuel Saxon, of Bruton, Somerset, to Alice Maud, second daughter of the Rev. J. C. Farmborough, Rector of Moreton Baggott, Warwickshire. (822)

### NOTICE TO CORRESPONDENTS.

All MSS., Letters, Books for Review, and other matters intended for the Editor should be addressed The Editor, "The Hospital," The Hospital Building, 28 & 29 Southampton Street, Strand, W.C.

The Editor cannot undertake to return rejected MS., even when accompanied by stamped directed envelope.

**Notice to Advertisers and Subscribers.**

All Advertisements, Orders for Copies of The Hospital, and Business Communications should be addressed to **THE MANAGER (Not to the Editor)**, The Hospital, 28 & 29 Southampton Street, Strand, London, W.C.

The Cost of Subscription, post free, is as follows:—Per week, 2½d.; per quarter, 2s. 8d.; per half-year, 5s. 3d.; per annum, 10s. 6d. Foreign:—Per annum, 15s. 2d., payable, in all cases, in advance.

**NOTICE.—Vol. XXIX. of "The Hospital" (October 1900, to March, 1901), in handsome cloth binding will shortly be on sale at the Office of this Journal, price, 7s. 6d. Cases for binding the Numbers contained in this Volume 1s. 6d. Index to Vol XXIX., 2½d., post free.**

**The Monthly Part for April is now ready. Price 6d., by post 8d.**

### BOOKS RECEIVED.

R. L. Sharland.

"The Scientific Roll." Conducted by Alexander R msay.

The Medico-Psychological Association.
"Organo-Therapeutics in Mental Diseases." By C. C. Easterbrook, M.A., M.D., M.R.C.P.Ed.

McCaw, Stevenson, and Orr.
"Thoughts on Rome and other Essays." By Conway Scott.

Religious Tract Society.
"The Witness of Great Englishmen against the Papacy." By J. Macaulay M.A., M.D.

The Medical Publishing Company.

"Cyclic Albuminuria." By G. A. Sutherland, M.D.

H. Virtue and Co.
"The Convalescent's Diet." By Lucy Helen Yates.

Sheapherd's Printing Works.
"The District Midwifery Case Book." By Margaret Caley.

Simpkin, Marshall, Hamilton, Kent and Co.
"The 'A 1' Cookery Book." By H. N. L.

J. B. Lippincott and Co.
"A Clinical Treatise on Fractures." By William Barton Hopkins, M.D.

### Scraps and Gleanings.

His Majesty the King has granted his Royal Patronage to the Richmond Royal Hospital.

Dr. P. L. Benson has been reappointed Medical Officer of Health for the Rural District of Buckingham.

At the annual meeting of the Aberdeen Dispensary it was stated that during the past ten years 54,426 patients had been treated in the Aberdeen Royal Infirmary, 15.504 in the Aberdeen Royal Hospital for Sick Children, and 107,467 in the Aberdeen Dispensary, making a total of 167,397, the total cost of treating whom was £112,000. A sum of £4,000 is needed to enable the liability incurred in the erection of the new maternity hospital in connection with the dispensary to be wiped off.

The report of the Committee of Management of the Tunbridge Wells General Hospital on the question of the new buildings, the revised statutes, &c., was accepted at the recent annual meeting. The report recommends that the present hospital site be retained, and that if satisfactory arrangements can be made some of the adjoining property shall be acquired. The Committee also recommend (1) that the accommodation be increased to provide for 76 beds, including a new ward for children; (2) a new out-patient department, and a new operating room be provided; (3) the accommodation for the nurses and servants be increased.

### The Student of Medicine

### APPOINTMENTS.

**A. J. Bowman Adams**, M.R.C.S., L.R.C.P., appointed Medical Officer for the Barnes District of the Richmond Union, and of the Children's Home, Barnes. Also, Divisional Surgeon Metropolitan Police, V. and Thames Divisions. **Robert Y. Aitken**, M.D.Glasg., M.R.C.S.Eng., L.R.C.P.Lond., appointed Honorary Assistant Surgeon at the Blackburn and East Lancashire Infirmary. **W. B. Anderton**, M.B., M.R.C.S., appointed Senior Resident Medical Officer to the Manchester Union Hospital, Crumpsall. **Marmaduke Bannister**, M.B.Lond., M.R.C.S.Eng., appointed Honorary Assistant Physician at the Blackburn and East Lancashire Infirmary. **W. H. Box**, M.R.C.S., appointed District Medical Officer of the Axbridge Union. **Michael Brick**, L.R.C.P., L.R.C.S.Irel., appointed Dispensary Medical Officer for the Tralee (No. 2) District. **A. D. Crawford**, M.B., C.M.Glasg., appointed Certifying Factory Surgeon for the Coleshill District of Warwickshire. **E. Cusse**, M.R.C S.Eng., appointed District Medical Officer of the Stockbridge Union. **Arthur Foster**, M.D.Edin., appointed Honorary Assistant Physician at the Blackburn and East Lancashire Infirmary. **Arthur E. Giles**, M.D., B.Sc., F.R C.S , M.R.C.P., appointed Gynæcologist to the Hospital of St. Francis, New Kent Road, S.E. **J. E. Judson**, M.R.C.S.Eng., L.R.C.P.Lond., appointed Second Resident Medical Officer to the Manchester Union Hospital, Crumpsall. **E. Laval**, M.B.Edin., appointed Second Resident Medical Officer to the Brislington House Private Asylum, near Bristol. **Charles G. S. Leeds**, M.B., L.R.C.P.Edin., appointed Assistant House Physician to the Wolverhampton and Staffordshire General Hospital. **L. R. Lempriere**, M.B., Ch.B., B.A., appointed Junior Resident Medical Officer to the Manchester Union Hospital, Crumpsall. **William Martin**, M.B., C.M.Glasg., appointed Assistant Surgeon to the Cardiff Infirmary. **P. W. Moore**, M.B.Lond., M.R.C.S.Eng., appointed District Medical Officer of the Shardlow Union. **Alex. W. Nuthall**, F.R.C.S., appointed Resident Surgical Officer of General Hospital, Birmingham. **J. M. A. Olivey**, M.R.C S., L.R.C.P.Lond., appointed District Medical Officer of the Poole Union. **T. E. Pallett**, M.D.Brux., L.S.A., appointed District Medical Officer of the Halstead Union. **R. W. C. Pierce**, M.B.Lond., D.P.H.Camb., appointed Medical Officer of the Joint Isolation Hospital of the Guildford Rural and Woking Urban Districts. **Clarence Read**, M.R.C.S., L.R.C.P., appointed an Honorary Officer to the North Shore Hospital, North Sydney, N.S.W. **John A. Rees**, M.B., C.M.Edin., appointed District Medical Officer of the Aberystwith Union. **Walter Rigby**, M.B., C.M.Edin., appointed Honorary Assistant Surgeon at the Blackburn and East Lancashire Infirmary. **Miss Marion J. Ross**, M.D.Glasg., appointed Junior House Surgeon to the Macclesfield Infirmary. **W. Scott Tebb**, M.D.Camb., D.P.H., appointed Medical Officer for the Penge Urban District. **John V. E. Tighe**, M.B., B.Ch., B.A.O.R.U.I., appointed Second Medical Officer to the North Riding Lunatic Asylum, Clifton, Yorks. **R. G. Worger**, M.R C.S., L.R.C.P.Lond., appointed Medical Officer for the Kilmersdon District of the Frome Union.

**THE MENOPAUSE AND ITS DISORDERS.**

With Chapters on Menstruation. By A. D. Leith Napier, M.D., M.R.C.P., late Editor of the *British Gynæcological Journal.* Royal 8vo. cloth gilt, illustrated by a series of Original Photo micrographs and many Illustrations in the Text. 7s. 6d. net.

"Of great practical use to the general practitioner as well as to those more specially engaged in gynæcological work."—*The Hospital.*

THE SCIENTIFIC PRESS, Ltd.,

28 and 29 Southampton Street, Strand, London, W.C.

